# The Failure to Measure Dietary Intake Engendered a Fictional Discourse on Diet-Disease Relations

**DOI:** 10.3389/fnut.2018.00105

**Published:** 2018-11-13

**Authors:** Edward Archer, Carl J. Lavie, James O. Hill

**Affiliations:** ^1^EvolvingFX, Jupiter, FL, United States; ^2^John Ochsner Heart and Vascular Institute, Ochsner Clinical School, The University of Queensland School of Medicine, New Orleans, LA, United States; ^3^Center for Human Nutrition at the University of Colorado, Aurora, CO, United States

**Keywords:** nutrition, diet, epidemiology, implausible, fallacy, category error

## Abstract

Controversies regarding the putative health effects of dietary sugar, salt, fat, and cholesterol are not driven by legitimate differences in scientific inference from valid evidence, but by a fictional discourse on diet-disease relations driven by decades of deeply flawed and demonstrably misleading epidemiologic research. Over the past 60 years, epidemiologists published tens of thousands of reports asserting that dietary intake was a major contributing factor to chronic non-communicable diseases despite the fact that epidemiologic methods do not measure dietary intake. In lieu of measuring actual dietary intake, epidemiologists collected millions of unverified verbal and textual reports of *memories of perceptions of dietary intake*. Given that actual dietary intake and reported memories of perceptions of intake are not in the same ontological category, epidemiologists committed the logical fallacy of “*Misplaced Concreteness.”* This error was exacerbated when the anecdotal (self-reported) data were impermissibly transformed (i.e., pseudo-quantified) into proxy-estimates of nutrient and caloric consumption via the assignment of “reference” values from databases of questionable validity and comprehensiveness. These errors were further compounded when statistical analyses of diet-disease relations were performed using the pseudo-quantified anecdotal data. These fatal measurement, analytic, and inferential flaws were obscured when epidemiologists failed to cite decades of research demonstrating that the proxy-estimates they created were often physiologically implausible (i.e., meaningless) and had no verifiable quantitative relation to the actual nutrient or caloric consumption of participants. In this critical analysis, we present substantial evidence to support our contention that current controversies and public confusion regarding diet-disease relations were generated by tens of thousands of deeply flawed, demonstrably misleading, and pseudoscientific epidemiologic reports. We challenge the field of nutrition to regain lost credibility by acknowledging the empirical and theoretical refutations of their memory-based methods and ensure that rigorous (objective) scientific methods are used to study the role of diet in chronic disease.

## Introduction

“*In an honest search for knowledge, you quite often have to abide by ignorance for an indefinite period. Instead of filing the gap by guesswork… however irksome the gap may be, its obliteration by a fake removes the urge to seek a tenable answer… The steadfastness in [this obligation], nay in appreciating it as a stimulus and a signpost to further quest, is a natural and indispensable disposition in the mind of a scientist*.” Erwin Schrodinger (1)

### The success of nutrition science

The science of diet-disease relations has a long, illustrious and successful history dating to antiquity (2). One of the first clinical trials ever conducted was an examination of the effects of citrus fruits on scurvy in the eighteenth century (3). And since that time, the science of nutrition achieved clinical and public health successes that were unimaginable in the distant past. For example, in the early twentieth century, diet-related diseases such as beriberi, rickets, and goiter were major public health challenges. In the United States (US), pellagra (a disease of niacin deficiency) caused the death of more than 100,000 Americans and afflicted more than 3 million (4). In New York City, 75% of all infants and nearly 100% of African American children suffered from rickets, a painful disease of vitamin D deficiency (5). Yet by the turn of the twenty-first century, diet-related diseases were almost non-existent and biochemical analyses demonstrated that ~80% of Americans (aged ≥ 6 years) were *not at risk of deficiencies* in any of the 7 vitamins examined (i.e., vitamins A, B-6, B-12, C, D, and E) (6, 7); and almost 90 percent of women of childbearing age (i.e., 12–49 years of age) were not at risk of iron deficiency, while folate levels increased ~50 percent since 1998 (6–8). Thus, the improvement of the US food supply and the “American Diet” over the past century was one of the greatest public health success stories in history (9).

#### Speculations and the shift to implausible anecdotal evidence

By the mid- twentieth century, as diet-related diseases (e.g., scurvy, pellagra) and protein-energy malnutrition became increasingly rare in industrialized nations, a small but influential group of investigators began speculating that complex chronic non-communicable diseases (NCDs) were somehow causally related to diet [e.g., see (10–14)] The evidence ostensibly supporting these conjectures was inferred from epidemiologic studies based on the naïve notion that a person's usual dietary intake (i.e., consumed foods and beverages) could be estimated simply by asking what he or she remembered eating and drinking (9, 15–17). Despite the fact that the data collected by these memory-based dietary assessment methods (M-BMs) were non-falsifiable anecdotes (i.e., verbal or textual self-reports) and were repeatedly demonstrated to lack validity and reliability as far back as the 1950s (18, 19), studies employing M-BMs came to dominate the empiric, policy, and media landscapes (15–17, 20). By the 1980s, tens of thousands of research reports based on M-BMs were published and some of these publications became the most highly-cited and widely publicized articles in the bio-medical literature.

Nevertheless, six decades of rigorous and highly-replicated research demonstrated unequivocally that M-BMs data were physiologically implausible (i.e., meaningless numbers) (9, 15–17, 21–25), often “*incompatible with life,”* (26) and had trivial relations to actual nutrient and caloric consumption (9, 15–17, 21–23, 26–44). For example, in 2013 we used multiple methods to show that from 1971 to 2010, no human being could survive on the average reported caloric intake in the National Health and Nutrition Examination Survey (NHANES) (21). Furthermore, when hypotheses derived from nutrition epidemiologic research were tested using rigorous study designs, they failed to be supported (45–49). For example, when over 50 nutrition claims were examined, “*100% of the observational claims failed to replicate”* and five conjectures were statistically significant “*in the opposite direction*” (50). These authors further stated “*the public at large–are being deceived, and are being deceived in the name of science. This should not be allowed to continue”* (50). While these authors wrote “*The cause is elusive*” (50), we showed that the refutation of diet-related hypotheses was due to the failure of nutrition epidemiologists to actually measure the variable of interest: dietary-intake.

This failure, in concert with the fact that the diet-disease associations did not meet Bradford Hill's criteria for causation (51), engendered a fictional discourse on the health effects of sugar, salt, fat, and cholesterol. This fictional discourse was widely disseminated by the popular press and exacerbated by governmental and non-governmental health organizations, and commercial interests (e.g., weight-loss industries and manufacturers of “heart-healthy” foods). The wide-spread publication of spurious diet-related speculations had significant adverse public health consequences (15, 20) that included public confusion (15, 52), regressive and misdirected public policy (20, 53, 54), the misallocation of research resources (9), scientifically illiterate recommendations on sugar (15, 55), and potentially harmful recommendations on salt (20, 56–60) that may have led to “*deaths due to hyponatremia”* (20) p. 22.

### Purpose of this critical analysis

Given the fictional discourse on diet-disease relations and the escalating debate over the validity of M-BMs (16, 17, 61–64), the purpose of this critical analysis is to present evidence that the current controversies regarding diet-disease relations are not driven by legitimate differences in scientific inference on the physiologic effects of dietary intake (i.e., consumed foods and beverages). Rather, we contend that current confusion on the putative health effects of dietary sugar, salt, fat, and cholesterol were engendered by a fictional discourse on diet-disease relations created by deeply flawed, demonstrably misleading, and pseudoscientific nutrition epidemiologic reports (9, 15–17, 20, 21, 23, 55). Herein, we argue that the confusion created by the fictional discourse and use of pseudoscientific methods to inform public policy led to the field of nutrition science losing credibility and scientific authority. Thus, to regain the public's trust, it is necessary for the field to acknowledge the empirical and theoretical refutations of M-BMs and ensure that in the future, rigorous scientific methods (e.g., randomized control trials, RCTs) are used to study the role of diet in chronic disease.

## The fatal flaws of nutrition epidemiology and the use of M-BMs

M-BMs, such as 24-h recall interviews (24 HR) and food frequency questionnaires (FFQs), are the predominant protocols in nutritional epidemiologic research. These methods continue to be funded and employed despite several facts and 60+ years of highly replicated, rigorous evidence that invalidate their use (9, 16, 17). In this critical analysis, we present this evidence and several arguments detailing the fatal flaws of M-BMs.

### M-BMs do not measure dietary intake

Most nutrition epidemiologists do not measure dietary intake (i.e., consumed foods and beverages) or the nutrient and caloric consumption of their participants. Instead, these investigators use M-BMs to collect unverified self-reported memories of what Willett in his popular textbook on nutritional epidemiology called “*perceptions of usual intake”* (65) p.111. Stated simply, the data collected via M-BMs are not quantitative estimates of dietary intake, but are mere guesstimates of whatever the respondents are willing and able to remember and report about what they think they ate or drank in the past, or want the investigators to think they consumed (9, 16, 17, 21, 23, 32, 37, 44). Thus, because the information collected is founded exclusively upon the respondent's honesty, memory, willingness, and ability to estimate and report past dietary intake (9, 15, 21, 23), investigators have no control over the quality, quantity, or error of the data collected via M-BMs.

Accordingly, there is a large disparity between anecdotal and objective evidence of dietary intake (9, 16, 17, 21–23, 26–44, 62). This disparity is largely explained by myriad intentional and non-intentional distorting factors, such as reactivity, confabulation, lying, forgetting, false memories, mis-estimation, and social desirability (9, 23, 32, 37). In rigorous scientific fields such as chemistry, biology, and physiology, anecdotal data are not used, and uncorroborated self-reported data are considered “inadmissible” as valid evidence (9). As such, we think that if nutrition epidemiologists wish to regain lost credibility, the use of M-BMs should be discontinued and not be used to inform public policy (9, 15, 20, 23).

#### Ten million jelly-beans: M-BMs collect numerically labeled qualitative data, not quantitative data

Verbal and textual reports of dietary intake are numerically labeled qualitative data (i.e., guesstimates). These data are not quantitative measurements of dietary intake. For example, if a person reports consuming ten million jelly beans, it should be apparent that this guesstimate is not a quantitative measure of actual food consumption but is merely a numerically labeled qualitative report (i.e., an anecdote). This remains a fact even if the person's guesstimate is more plausible (e.g., 50 jelly beans) because neither self-report was based on an objective measurement protocol. Therefore, without objective corroboration these “data” are not falsifiable and therefore must be considered pseudo-scientific (9, 16, 17, 22, 23).

#### Human memory and recall are not valid tools for data collection

Human memory and recall are not valid tools for scientific data collection (9), and it is well-established that subjective reports of past events (i.e., anecdotes) are not representative of objective facts (9, 66–70). There is a vast body of evidence demonstrating that “*one fact stands out more than any of the others—the very worthlessness of human testimony…”* (71) p.13–14. For example, after reviewing the validity of self-reported data in nutrition, health-care, anthropology, communications, criminal justice, economics, and psychology, over three decades ago Bernard et al., concluded “*on average, about half of what informants report is probably incorrect…*” (66).

#### Deception: people lie when asked to report their dietary intake

Deception is an inherent part of human nature and the majority of people intentionally misreport (i.e., lie) when reporting their dietary intake (16, 17, 29, 30, 72). For example, when asked to report their dietary intake, 78% of clinical and 64% of non-clinical participants “*declared an intention to misreport”* (37) p. 209. This is a common finding (39, 41, 72), yet epidemiologists make no attempt to address the reality of intentional misreporting in their research. This failure is a major contributor to the fictional discourse on diet-disease relations because the participants' actual dietary intake is unknown and what is reported may be “pure” fiction (73).

#### Reactivity: participants alter behavior

It is well-established that participants alter their consumption when asked to report their dietary intake (29, 31, 35, 37, 39, 41). For example, when asked, participants offer socially desirable reports based on current dogma, such as consuming less “*fatty foods…*[and consuming more]…*fruits and vegetables”* (30) p. 792. This body of work unequivocally demonstrates that self-reported dietary data are not representative of usual dietary intake, and therefore contribute to the fictional discourse. Yet more importantly, given that health-conscious participants are more likely to engage in exercise, not smoke, and report their dietary intake in a manner consistent with current dietary dogma (e.g., eat more fruits and vegetables), current nutritional epidemiologic studies simply reinforce past recommendations without providing any information regarding the validity of those recommendations. Defenders of M-BMs fail to grasp the significance of this bias (i.e., dietary recommendations are a self-fulfilling prophecy) and often cite the allegedly positive effects of past dietary recommendations as support for the validity of M-BMs [e.g., see (62, 74–76)].

#### Credulousness is antithetical to scientific data collection

It is unequivocal that a person's reported memories of perceptions of a past event are not an accurate qualitative or quantitative representation of that event (68, 70, 77, 78). This reality, in confluence with the fact that people often misrepresent their perceptions to influence the opinion of others, demonstrates that reported memories of perceptions of consumed foods and beverages are not valid. In a scathing critique of self-reported data, Lewontin wrote, “*It is frightening to think that…science is in the hands of professionals so deaf to human nuance that they believe that people do not lie…and that they have no interest in manipulating the impression that strangers have of them”* (79) p. 28. Nevertheless, nutrition epidemiologic investigators treat their participant's anecdotes (i.e., dietary self-reports) as accurate and truthful despite voluminous evidence to the contrary (9, 16, 17, 21, 23). Thus, given that blind-faith is antithetical to scientific data collection, the credulousness of epidemiologists, and their failure to account for basic human behaviors (e.g., deception and reactivity) were major drivers of the fictional discourse on diet-disease relations.

### M-BMs are founded upon a “category error” and the logical fallacy of “misplaced concreteness”

#### Category error and incommensurability

A “Category Error” is an ontological mistake. The standard exemplar is the conflation of abstract phenomena with physical objects (80). Mental phenomena, such as memories and perceptions, do not exist outside the mind of the perceiver and therefore cannot be observed nor measured. Conversely, physical objects (e.g., foods and beverages) exist independent of the perceiver, and are therefore, observable, and potentially measurable. Thus, actual dietary intake and memories of perceptions of dietary intake are distinct and incommensurable (i.e., nonequivalent) and a large body of rigorous evidence demonstrated this non-equivalence (9, 21–23, 26–44, 62). Thus, when employing M-BMs, nutrition epidemiologists are attempting to “*measure the unmeasurable”* (16) and continue to commit a fatal “Category Error” by erroneously assuming that reported memories of “*perceptions of usual intake”* (65) p. 50 are the equivalent of actual dietary intake.

#### Reification: “misplaced concreteness”

Reification is the fallacious practice of treating abstract entities as if they were physical objects and is the behavioral extension of a “Category Error.” For example, memories of perceptions of dietary intake are mental phenomena about food and beverages that may or may not have been consumed in the past (9, 68, 81). It should be obvious that these purely mental phenomena do not, and cannot, contain nutrients or calories. Thus, when epidemiologists assign nutrient and caloric values to memories of perceptions of dietary intake they are “*mistaking the abstract for the concrete*” (82) p. 73. Therefore, when verbal or textual reports of memories of perceptions of dietary intake are presented as estimates of actual intake or nutrient and caloric consumption, these data are both pseudo-scientific and misleading (9, 20).

### M-BMs data rely on pseudo-quantification

Self-reported perceptions of consumed foods and beverages are not estimates of nutrient or caloric consumption. Therefore, statistical analyses of diet-disease relations require the creation of proxy-estimates of nutrient and caloric consumption via the *post-hoc* assignment of reference nutrient and energy values to the verbal or textual reports. This process is known as pseudo-quantification because it transforms qualitative (i.e., nominal/anecdotal) self-reports into quantitative (i.e., ratio) data via the assignment of numerals. As will be discussed in the following section, pseudo-quantification differs from scientific measurement because measurement is the discovery, not assignment of numeric relations (83–85).

#### Pseudo-quantification: the converse of scientific measurement

Scientific measurement is the empirical process of discovery in which a known unit of an observable phenomenon (e.g., a milligram of Vitamin C) is compared to the magnitude (i.e., amount) of that unit in an observable entity (e.g., an orange). The mere assignment of numerals is not the scientific equivalent of measurement because the assigned number may or may not have an empirically supported relation to the observed entity (83, 84). Nevertheless, rather than measuring the actual consumption of participants, epidemiologic investigators merely *assign* “reference” nutrient and caloric values to self-reported foods and beverages to create proxy-estimates of consumption. This process of pseudo-quantification is literally the converse of scientific measurement because the actual nutrient and caloric consumption of the participants are never “discovered” and remain unknown.

#### Impermissible transformation: nominal data + assigned numerals ≠ quantitative data

Valid inferences on diet-disease relations necessitate measuring both consumed foods and beverages, and concomitant nutrient and caloric intake. M-BMs measure neither. The mere assignment of numerals to anecdotal evidence does not transform the qualitative (nominal) reports into quantitative (ratio) data. Thus, the process of pseudo-quantification is the impermissible transformation of incommensurable phenomena (i.e., converting abstractions into concrete entities). As described in the prior “Jelly-Bean” example, numerically labeled verbal or textual reports are not the equivalent of quantitative data obtained from measurement protocols. As such, there is no verifiable relation between the dietary reports and the actual foods and beverage consumed. More importantly, given the lack of validity and comprehensiveness of nutrient databases (as discussed below), there are no verifiable relations between the assigned nutrient and caloric values and the actual nutrient and caloric consumption of the participant. These fatal conceptual and measurement flaws explain why the tens of millions of extremely precise proxy-estimates created by epidemiologists over the past six decades were repeatedly demonstrated to be physiologically implausible (i.e., meaningless numbers) (9, 21, 23–25).

### Nutrient database issues

The databases used for the pseudo-quantification of FFQs and 24HRs, such as the National Health and Nutrition Examination Survey (NHANES), contain <8,000 unique foods (86). Yet it was estimated that more than 85,000 unique items exists in the ever-expanding US food supply (86) and over 200,000 unique food codes were published in the US Department of Agriculture's (USDA) Food Composition Databases (24, 87). Thus, given that FFQs collect “*a finite list of foods/portions with little detail*” (62) p. 2 and include only 75–200 items, it is highly unlikely that the extremely precise nutrient and caloric values assigned to FFQ or 24HR data are representative of what was actually consumed (16, 17, 24, 25). Given these facts, both FFQs and 24-HRs lack face validity (16, 17).

Importantly, unlike the standard coefficients used in chemistry, standardized reference nutrient or caloric values for foods and beverages do not exist. Inherent variability and the “*rapidly changing landscape of the food supply”* (86) prevent the creation and publication of accurate reference values. For example, variation in farming practices (e.g., seed and soil quality), as well as time, temperature, and other changes induced via storage and processing (e.g., cooking), and industry reformulations render the accuracy and reliability of published reference values uncertain, if not wholly invalid (86, 88, 89). For example, Merchant et al. stated, “*there may be large discrepancies in nutrient content of foods between the USDA database and what is found in the field because of manufacturing practices*,” (90) p. 7 and Phillips et al., stated, “*Reliable methods are simply not currently available for some components in all foods (e.g., folate, Vitamin D)”* (91) p. 1354. Similarly, Deharveng et al. stated, “*…due to the high natural variation in foods…*[and]…*the use of several sources which may mean that the nutritional values are not comparable within the same table…*[and] “*common methods and definitions…or modes of expression (energy, protein, carbohydrates, carotenes, vitamin A and E) have not yet been agreed upon, so values are not comparable* [between or within databases, and there are] …*values produced over 20 years ago with outdated analytical methods”* (92) p.60. Moreover, there are numerous nutrient databases with varying degrees of accuracy (e.g., incorrect values) and completeness (e.g., missing values) (86, 88, 90, 92–99), and the investigators' choice of database affects results and conclusions (89, 91, 92, 100). As Natarajan et al., wrote, “*self-report measures could be strongly biased by the inherent errors in the nutrient databases' ability to estimate true…intake*” (100) p. 776.

These results demonstrate there can be no standardized or valid “reference” caloric and nutrient values. As such, the pseudo-quantification of self-reported memories, digital images of foods and beverages, or other recent advances [e.g., “eating” sensors and instrumented utensils (101)] offer no valid or verifiable quantitative information on the actual caloric or nutrient consumption of participants. Therefore, there are no valid nutrient and energy data from which to examine diet-disease relations. Thus, pseudo-quantification is a major driver of both the implausibility of M-BM data and the fictional discourse on diet-disease relations.

### Implausible proxy-estimates of nutrient and energy consumption

The process of pseudo-quantification does not produce accurate or even believable proxy-estimates of nutrient or caloric consumption. Six decades of rigorous research demonstrated unequivocally that the proxy-estimates created by investigators were often physiologically implausible (i.e., meaningless numbers), (9, 21, 23–25) “*incompatible with life”* (26) p. 347, and have trivial relations to actual nutrient and caloric consumption (9, 21–23, 26–44, 62). For example, severe, systematic, and *intentional* under- and over-reporting of specific foods and beverages (e.g., sugar, vegetables, alcohol), and absolute caloric and nutrient consumption (e.g., protein, sodium) are omnipresent (9, 21–23, 26–44, 62). These highly-replicated results demonstrate that any conclusions regarding diet-disease relations inferred from M-BM-based research are not valid.

### The non-quantifiability of measurement error

#### Falsifiability: discerning fact from fiction

The discrimination between scientific and pseudoscientific (i.e., non-falsifiable) data is contingent upon the ability to discern fact from fiction. To accomplish this task in nutrition, it is first necessary to ascertain if the reported foods and beverages match the respondent's actual intake; and second, it is necessary to quantify the disparity between the proxy-estimates created via pseudo-quantification and the respondent's actual caloric and nutrient consumption.

Thus, accurate estimations of the types and amounts of consumed foods and beverages is required. Yet, as we demonstrated previously (9), this is not possible because “*without objective corroboration it is impossible to quantify what percentage of the recalled foods and beverages are completely false, grossly inaccurate, or somewhat congruent with actual consumption”* (9) p. 919 and “*neither the researchers nor the participants know the validity or reliability of the reported food and beverage consumption…”* (9) p. 918. Similarly, it is not possible to quantify the disparity between the proxy-estimates of nutrient and caloric consumption and the respondent's actual consumption because the nutrient and caloric intakes of participants were never measured; these values were merely *assigned* to the reported foods and beverages. Importantly, the errors in the original verbal or textual reports will be propagated unpredictably via pseudo-quantification in a non-quantifiable manner. This renders the final estimates of nutrient and caloric consumption essentially meaningless. Thus, statements that 24 HR, “*have known measurement errors…”* (102) p. 922 and that 24HR data can be “*adjusted for total energy* [to] *reduce measurement error”* (103) p. 2552 are false and contribute to the ongoing fictional discourse on diet-disease relations.

In summary, M-BMs cannot estimate the participants' true consumption of foods and beverages. Therefore, the participants' actual nutrient and caloric intake are unknown and unknowable; and because the measurement error associated with M-BMs is non-quantifiable and non-falsifiable, self-reported dietary data are inadmissible as scientific evidence (9, 23). Thus, six decades of statistical analyses of pseudo-quantified (i.e., reified) dietary anecdotes were a major contributor of the fictional discourse on diet-disease relations (15–17, 55).

## Misleading publications and the fictional discourse on diet-disease relations

### Failure to cite contrary evidence

Over the past six decades, epidemiologists published tens of thousands of research reports in which millions of self-reported memories of perceptions of dietary intake were presented as the equivalent of data on the actual dietary intake of participants. Nevertheless, and despite a century of research from multiple domains (e.g., psychology, sociology, and cognitive neuroscience) demonstrating that this presentation was patently false and misleading (9, 16, 17, 66–70), epidemiologists often failed to cite, acknowledge or address the overwhelming contrary evidence.

For example, in 2013, we demonstrated via multiple methods that over the past five decades the average caloric intake reported in the NHANES could not support human life (21) and that >40% of NHANES participants' reported caloric intakes were below the level needed to support a comatose patient's survival (9, 21, 23, 104). Yet despite the clear refutations and empirically supported rebukes of M-BMs, the 2015 Dietary Guidelines Advisory Committee (DGAC) (105) falsely wrote that the implausible NHANES data, “*provide national and group level estimates of dietary intakes of the U.S. population, on a given day…*” (105) Part C, p 13, Similarly, the Dietary Guidelines for Americans (DGA) presented these implausible (i.e., meaningless) NHANES dietary data as “*Current Eating Patterns in the United States*.” (106) Chapter 2.

Furthermore, 80% of the studies in the US Department of Agriculture's National Evidence Library (107) used by the 2015 DGAC to establish the DGA employed M-BMs. We contend that the unremitting use of this large body of refuted evidence in concert with the failure to cite or even acknowledge contrary evidence exacerbated the fictional discourse and led to the “*Disease-Mongering of the American Diet”* (108) and the “demonization” of dietary sugar (15, 55). For example, despite biochemical analyses demonstrating that the vast majority of Americans were not at risk of vitamin and mineral deficiencies (7), the 2015 DGAC report stated that “*several nutrients are underconsumed*”18(PartA,p20) (i.e., vitamins A, C, D, E, and folate) and that for women of childbearing age, “*iron also is a shortfall nutrient*.”18(PartA,p2). Clearly Americans could not have adequate serum levels (as demonstrated by biochemical analyses), if these vitamins and minerals were “underconsumed.”

The reason for the apparent underconsumption of minerals and vitamins was the well-established fact that self-reported dietary intakes are severely and systematically under-reported via M-BMs. Thus, by failing to acknowledge the existence of contrary biochemical data and reporting only the implausible NHANES M-BM data, the 2015 DGAC presented a false and alarmist perspective on the nutritional status of Americans. This misleading presentation was an exemplar of “*disease-mongering”* (15, 108) because it distorted the scientific record and misled both the public and policy makers by erroneously suggesting that Americans were at risk for nutritional deficiencies when objective evidence demonstrated they were not (15, 55).

More recently, the National Academies of Sciences, Engineering, and Medicine examined the process by which the Dietary Guidelines for Americans were created. Their report, “*Redesigning the Process for Establishing the Dietary Guidelines for Americans”* (109) was ostensibly intended to review the ongoing controversy surrounding the rigor and validity of the evidence employed. Nevertheless, the authors failed to cite any of our numerous peer-reviewed publications refuting the validity of the NHANES dietary data, and in direct opposition to extant evidence, the authors of the report wrote, “*After extensive evaluation, we found that the current methods being used in the DGA process…are indeed appropriate”* (109) p. x; Preface [and] “*Self-report dietary intake* [M-BMs] *data are central to the development of dietary guidelines”* (109) p. 4–13. Thus, by summarily excluding our large body of contrary evidence and failing to inform readers of the empiric, theoretic and conceptual refutations of their methods and data, the National Academies of Sciences, Engineering, and Medicine exacerbated the fictional discourse on the putative health effects of dietary sugar, salt, fat, and cholesterol. This biased presentation has significant public-health consequences (e.g., public confusion, ineffective, and regressive public policy, and misallocation of research resources) (9, 15, 20).

### Recent contributions to the fictional discourse on diet-disease relations

Most recently, the Journal of Clinical Epidemiology published a series of “Controversy and Debate” articles on the “*Fatal Flaws of Food Frequency Questionnaires*…” (16, 17, 63, 64). In our target paper (17), we presented a number of very specific challenges to the status quo in nutrition epidemiology. Nevertheless, our esteemed opponents in the debate failed to address the issues and chose to offer mere ipse dixit statements and fallacious arguments (e.g., *ignoratio elenchi, ad hominems, ad populum*) (63, 64). Thus, in our closing statement we wrote that improving nutrition science and public health policy will be achieved only if the epidemiologic research community acknowledges and addresses contrary evidence and empirical refutations (16).

## Challenges and relevancy of estimating dietary intake

### Challenges to measuring or estimating dietary intake

Given that deception and reactivity are inherent components of human relations, acquiring accurate information on behaviors that are subject to social approbation or stigma is extremely challenging. Thus, it is highly unlikely that data derived from uncorroborated self-reports and other forms of information controlled exclusively by the participant will ever be valid. For example, while there is emerging interest in digital food imaging, bite counters, instrumented utensils and other movement sensors to estimate dietary intake (101, 110, 111), it should be apparent that these technology-based methods do not address the major issues presented herein (e.g., deception, reactivity, pseudo-quantification, and invalidity of reference nutrient and energy values). For example, individuals wishing to “game” the system can merely remove the devices when eating or take digital images of their dining companion's salad while consuming a high-calorie dessert. Thus, despite their novelty and inventiveness, these technological developments currently offer little progress in estimating habitual dietary consumption because they do not account for basic human behaviors (e.g., intentional deception and reactivity).

### Diet-centrism: “let food be thy medicine…”

Diet-disease relations were posited since antiquity and it has been asserted for millennia that individuals should “*Let food be thy medicine and medicine be thy food*” (112). While this ancient advice was relevant to individuals consuming nutritionally inadequate diets, for individuals and populations consuming biochemically superior diets [e.g., the average US citizen (6–8)] this advice is archaic and misleading (15).

Recently, we coined the term “Diet-Centrism” to describe the “*the naïve tendency of both researchers and the public to attribute a wide-range of negative health outcomes exclusively to dietary factors while neglecting the essential and well-established role of individual differences in nutrient-metabolism*” (15). As we demonstrated, the explicit conflation of “diet” with both nutritional status and health ignores the fact that the human body is a complex physiologic system in which dietary factors are merely one of myriad factors that affect health. And more importantly, the effects of dietary intake are entirely dependent on the physiologic context of the consuming individual (15). Thus, with respect to diet-related health, it is not what is eaten that affects health and disease, but what one's body does with what was eaten (113, 114). Therefore, as we previously detailed (15, 55, 113), the idea that “you are what you eat” is demonstrative of prescientific thinking [i.e., magico-religious reasoning (55)], “physiologic illiteracy” (15, 114), and flouts centuries of progress in medical science because dietary components cannot have effects independent of the physiologic context of the consuming individual (15, 55, 113, 114).

### Does “diet” have a non-trivial impact on health?

Modern scientific investigations established that if a person habitually fails to consume sufficient calories or protein to meet metabolic demands, that person will die due to protein-energy malnutrition (i.e., starvation). Similarly, if a person fails to consume adequate levels of nutrients, then he or she will suffer diseases specific to the dietary deficiency (e.g., scurvy from insufficient Vitamin C). Nevertheless, it is extremely important to note that in general populations the established *causal* effects of “diet” are limited to protein-energy malnutrition and nutrient deficiencies.

Contrary to current conjectures, there are no valid data demonstrating that “diet” *per se* is *causal* to increased mortality from obesity, NCDs, and metabolic diseases (15, 113). First, diet-centric speculations based on mere statistical associations provide no evidence of causation, and when tested via rigorous (i.e., objective) methods these hypotheses were repeatedly demonstrated to be false (50, 115, 116). Second, diet-disease conjectures on sugar, salt, and fat consumption fail to meet Bradford Hill's criteria for causality (e.g., strength, consistency, biological gradient, and specificity) (15, 51, 113). Third, the vast majority of diet-disease relations rely upon the validity of M-BMs. If as demonstrated herein, M-BMs are not valid, then ~80% of the research in the US Department of Agricultures' National Evidence Library (107) and most diet-centric speculations have no valid empirical support. Fourth, clinical trials examining intermediate biomarkers (e.g., serum lipids) and other surrogate risk factors are often irrelevant to the actual risk of mortality and morbidity (117). For example, it is well-established that while biomarkers may reflect the short-term physiologic effects of an intervention, these alterations are not necessarily indicative of changes in risk (118, 119). Fifth, myriad paradoxes [e.g., Australian, French, Finish, Irish, Israeli, Indian, Spanish, Masai, and Japanese (120–128)] and rigorous analyses (15, 113, 129, 130) suggest that sugar, salt, and fat are merely necessary for health and well-being but have no major impacts on chronic diseases (15, 113). For example, there are populations that consume as much as 80% of their caloric intake from added sugars with no obesity and metabolic diseases. For a review please see (15, 113). Finally, research dating to the 1950s demonstrated that the “over-consumption” that leads to obesity and metabolic disease is not driven by dietary factors *per se*, but by physical inactivity-induced increments in energy intake (113, 114, 131–134) and non-genetic evolutionary processes (i.e., accumulative maternal effects) (113, 114, 135–137) that lead to the asymmetric and adipogenic partitioning of nutrient-energy (114).

Thus, we posit that while dietary intake is an obvious and essential component of health, it is a trivial risk factor for obesity, metabolic, and chronic diseases (15, 55, 73, 113, 114). Our position is rapidly acquiring support given the “*tiny*” effect sizes and “*massive confounding*” inherent in nutrition research (61, 138–140). For example, when compared to the relative risk estimates of smoking tobacco, estimates for dietary factors are an order of magnitude smaller (140). In fact, these estimates are so trivial, “*crude and imprecise”* that most diet-disease associations may be considered spurious (141). As such, we posit that measuring “diet” *per se* is tangential if not irrelevant to the major public health issues faced by industrialized nations (113, 114).

## Forward progress

Because identical diets consumed by different individuals result in divergent metabolic and health effects (15, 114, 142–144), measuring “diet” without accurate and detailed knowledge of the metabolic phenotype of the consuming individual is pointless. And given that accurate metabolic phenotyping in large samples is prohibitively expensive in terms of resources and participant burden, valid epidemiologic (i.e., population-level) investigations of diet-disease relations may simply be unachievable.

Nevertheless, the metabolic fate of consumed foods and beverages are accessibly to quantification via laboratory settings because all dietary components are fully metabolized or excreted in experimentally relevant time-frames (i.e., minutes to days). Thus, the non-trivial physiologic effects of dietary intake can be ascertained via RCTs. Given this reality, it is important to note that the notion that “diet” has long-term physiologic or health effects independent of the metabolic fate of consumed bio-active molecules is a form of magico-religious reasoning (15, 55) and an impediment to scientific progress.

We realize that some may perceive our conclusions as both contrary and controversial. Nevertheless, we posit that the decades-long fictional discourse on the effects of dietary sugar, salt, and fat led to an extreme form of diet-centrism that obscured well-established evidence and engendered the proliferation of misleading and demonstrably false research programs and failed public health initiatives (15, 20, 113). Thus, given the evidence presented herein, it is incumbent upon nutrition epidemiologists to provide valid scientific support for their “diet-centric” speculations and demonstrate that the average “Western diet” (6, 7) has non-trivial effects on obesity and NCDs in industrialized nations (114).

## Summary and conclusions

Since its first clinical trial in the eighteenth century, the field of nutrition relied on the observable effects of an individual's dietary intake on his or her health. This scientific process led to the elimination of diet-related deficiencies and substantial improvements in public health. Nonetheless, beginning in the 1950s, the field allowed rigorous research to be obscured by the sensational but implausible results and conclusions generated by the pseudo-quantified anecdotal data generated via M-BMs. The devolution from rigorous scientific observation to anecdotal (i.e., self-reported) evidence led to a fictional discourse on diet-disease relations that resulted in both public and policy confusion, and a major loss of credibility for the nutrition sciences. We challenge the field to acknowledge the inherent flaws and empirical and theoretical refutations of M-BMs, and ensure that in the future, rigorous scientific methods (e.g., RCTs) are used to study the role of diet in chronic disease.

## Author contributions

All authors listed have made a substantial, direct and intellectual contribution to the work, and approved it for publication.

### Conflict of interest statement

EA is employed by EvolvingFX. The remaining authors declare that the research was conducted in the absence of any commercial or financial relationships that could be construed as a potential conflict of interest.
